# Anti-proliferative activity of silver nanoparticles

**DOI:** 10.1186/1471-2121-10-65

**Published:** 2009-09-17

**Authors:** PV AshaRani, M Prakash Hande, Suresh Valiyaveettil

**Affiliations:** 1 Department of Chemistry, Faculty of Science, 3 Science Drive 3, National University of Singapore, 117543, Singapore; 2 Department of Physiology, Yong Loo Lin School of Medicine, 2 Medical Drive, National University of Singapore, 117597, Singapore

## Abstract

**Background:**

Nanoparticles possess exceptional physical and chemical properties which led to rapid commercialisation. Silver nanoparticles (Ag-np) are among the most commercialised nanoparticles due to their antimicrobial potential. Ag-np based cosmetics, therapeutic agents and household products are in wide use, which raised a public concern regarding their safety associated with human and environmental use. No safety regulations are in practice for the use of these nanomaterials. The interactions of nanomaterials with cells, uptake mechanisms, distribution, excretion, toxicological endpoints and mechanism of action remain unanswered.

**Results:**

Normal human lung fibroblasts (IMR-90) and human glioblastoma cells (U251) were exposed to different doses of Ag-nps *in vitro*. Uptake of Ag-nps occurred mainly through endocytosis (clathrin mediated process and macropinocytosis), accompanied by a time dependent increase in exocytosis rate. The electron micrographs revealed a uniform intracellular distribution of Ag-np both in cytoplasm and nucleus. Ag-np treated cells exhibited chromosome instability and mitotic arrest in human cells. There was efficient recovery from arrest in normal human fibroblasts whereas the cancer cells ceased to proliferate. Toxicity of Ag-np is mediated through intracellular calcium (Ca^2+^) transients along with significant alterations in cell morphology and spreading and surface ruffling. Down regulation of major actin binding protein, filamin was observed after Ag-np exposure. Ag-np induced stress resulted in the up regulation of metallothionein and heme oxygenase -1 genes.

**Conclusion:**

Here, we demonstrate that uptake of Ag-np occurs mainly through clathrin mediated endocytosis and macropinocytosis. Our results suggest that cancer cells are susceptible to damage with lack of recovery from Ag-np-induced stress. Ag-np is found to be acting through intracellular calcium transients and chromosomal aberrations, either directly or through activation of catabolic enzymes. The signalling cascades are believed to play key roles in cytoskeleton deformations and ultimately to inhibit cell proliferation.

## Background

The convergence of nanotechnology with nanomedicine has added new hope in the therapeutic and pharmaceutical field. The unique nature of nanoparticles is being exploited by scientists, in hope of developing novel diagnostic and antimicrobial agents [1]. Silver nanoparticles (Ag-np) are widely used in medicine, physics, material sciences and chemistry [2]. However, the rapid progress in nanotechnology was accompanied by insufficient data on biohazard identification. Exposure to nanomaterials occurs through inhalation, ingestion, injection for therapeutic purposes and through physical contact at cuts or abraded skin. From the site of deposition, the nanoparticles are translocated to different parts of the body through the circulatory or lymphatic system. *In vivo *studies showed that exposure to nanoparticles could result in inflammation, oxidative stress, myocardial infarction and thrombosis [3]. In addition, a few of the nanoparticles can alter the permeability of blood brain barrier [4]. Although these concepts are applicable to nanomaterials in general, a detailed study is necessary for each nanoparticle in use.

Ag-nps are classified as antimicrobial agents [5,6] and are widely used in treating wounds, burns and catheter related infections [7]. Even though silver complexes were used for decorating sweets, as components of dental alloys [8] and antimicrobial agents in ancient times, lack of information regarding the toxicity remained an important issue. A few reports demonstrated the toxicity of silver nitrate on human dermal fibroblasts [9] and on aquatic species [10]. We had identified *in vivo *targets of silver nanoparticle in zebrafish embryos [11]. Silver nanoparticles were found to be deposited in various organs in zebrafish embryos giving rise to distinct developmental defects. Recent reports on Ag-np toxicity have identified the mitochondria [12] as primary targets of silver nanoparticles in rat liver cells. Moreover, Ag-nps were reported to act via reactive oxygen species (ROS) production and glutathione depletion in rat liver cells [12]. The recent work by Skebo and colleagues [13] claimed that Ag-nps coated with polysaccharide exhibited less cytotoxicity compared to other Ag-nps employed for the study.

Ag-nps are believed to alter the membrane structure by attaching to the sulphur containing proteins of the cell membrane thereby damaging the cell membrane of the bacteria [14,15]. Recent reports emphasised on the size and shape dependent interactions of Ag-nps with bacterial cells, which played a crucial role in their bactericidal properties [16]. The occupational hazard associated with the nanoparticle exposure and the molecular mechanisms underlying Ag-np toxicity are still unknown. Here, we set to investigate the mechanism of Ag-np toxicity by employing various techniques like fluorescence *in situ *hybridisation (FISH) to detect the chromosomal abnormalities and real-time reverse transcriptase polymerase chain reaction (RT-PCR) for monitoring change in gene expression patterns occurred due to nanoparticle treatment. The uptake and exocytosis rates of nanoparticles were estimated. Also, the Ca^2+ ^transients in the nanoparticle treated cells were monitored and the propagation of Ag-np signals was identified. A model is proposed based on the data which might explain the mechanism of Ag-np toxicity.

## Results

### Cellular uptake and exocytosis of nanoparticles

**Figure 1:**
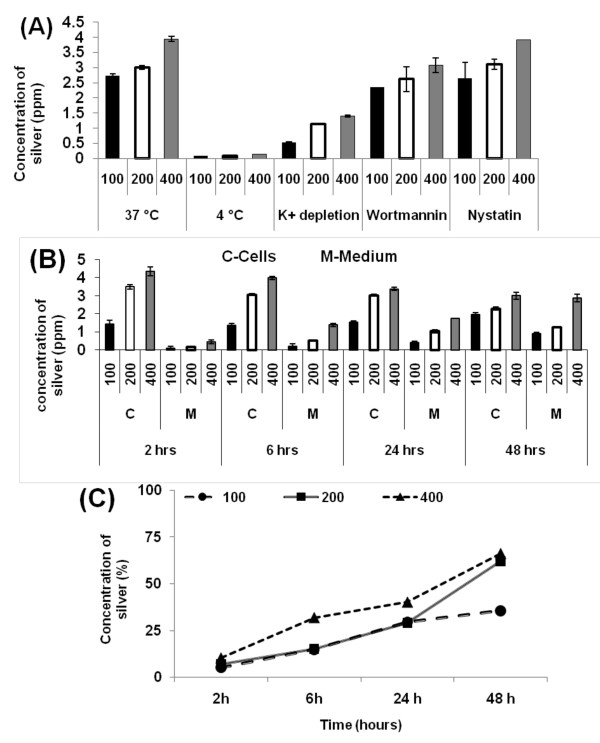
**Concentration of silver estimated using ICP-OES of Ag-np treated cells**. (**A**) Data obtained from endocytosis studies using U251 cells. X axis represents different conditions employed viz. K+ depletion and temperature. Cells incubated at 37°C showed double the concentration of Ag than cells incubated at 4°C and K+ depleted environment. The concentration of Ag isolated from clathrin blocked cells and endocytosis blocked cells were approximately equal illustrating a clathrin independent process of uptake. (**B**) Exocytosis data show a concentration and time dependant exocytosis as indicated by the increased concentration of Ag in culture supernatant and a gradual decrease in cells. X axis represents different time and concentrations employed for exocytosis. Y axis represents concentration of silver detected by ICP-OES. (**C**) Comparison of exocytosis and endocytosis rate in U251 cells. Percentage of endocytosed nanoparticles expelled from cells over a period of time is depicted in the graph.

Attempts to identify the uptake routes of Ag-np led to the conclusion that Ag-np were taken up mainly through endocytosis. Clathrin dependent endocytosis and macropinocytosis played major role in Ag-np uptake. Untreated cells showed no detectable levels of silver. The cells incubated at low temperature had much reduced concentration of silver (less than 1 ppm) as compared to 37°C incubated cells, which implies that endocytosis is the major pathway for Ag-np uptake (Figure 1A). Nevertheless, inhibition of clathrin pits formation did not result in a complete drop in endocytosis which indicates that Ag-np endocytosis can occur in the absence of clathrin pits. When cellular uptake through macropinocytosis was aborted, a significant drop in Ag-np uptake was observed, suggesting the active involvement of the pathway. Inhibition of caveoli pits did not affect Ag-np uptake. Exocytosis studies showed a gradual increase in silver concentration in cell culture supernatants together with a drop in nanoparticle concentration in cell homogenates. The nanoparticle concentration in culture medium showed a time and concentration dependant increase (Figure 1B). The process of exocytosis was compared with the endocytosis by calculating the percentage of drop of total endocytosed silver to that of exocytosed with time (Figure 1C). The exocytosis followed a concentration dependant increase over time, where ~ 66% of endocytosed nanoparticles where exocytosed by 48 hours compared to an exocytosis of ~ 10% at 2 hours. A long incubation period of 48 hours was taken to expel 66% of the particles endocytosed in 2 hours, implying a slow exocytosis rate than endocytosis. A significant amount of endocytosed nanoparticles (34%) was still retained by the cells.

### Recovery and colony formation

**Figure 2:**
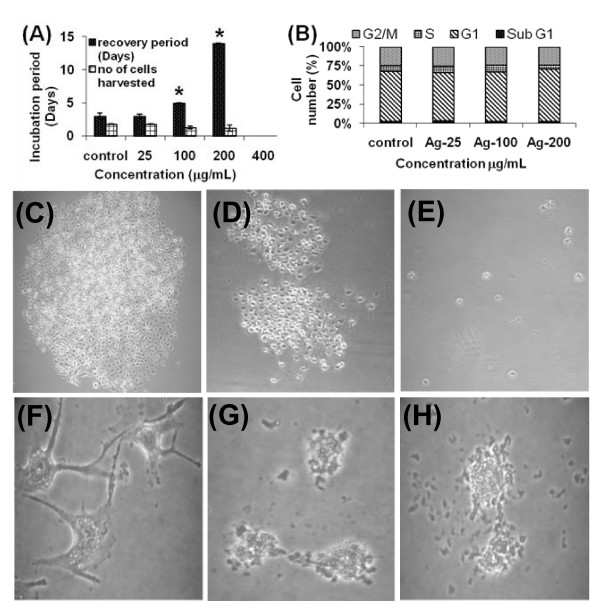
**Recovery studies**. Colony formation studies of Ag-np treated IMR-90 and U251 cells: The cells after Ag-np exposure were allowed to recover from stress. The time in days taken for the cells to reach confluence is expressed as the recovery period (Y-axis) (A). Concentrations of nanoparticles are indicated on the X- axis. Cells treated with all concentrations except 400 μg/mL recovered. *p < 0.05. (**B**) Cell cycle analysis of recovered cells showing absence of cell cycle arrest (n = 3). (**C**) Untreated cancer cells showing recovery and (**D**) Ag-np treated (25 μg/mL) cells forming colonies (1 week). (**E**). Cells treated with higher concentration of Ag-np (200 μg/mL) did not form colonies. The morphology of the cells under recovery deteriorated with time. (**F**) Control cells with proper protoplasmic extensions. (**G**) Ag-np treated cancer cells showing unhealthy cells with no proper protoplasmic extensions (2 weeks). (**H**) Morphological deterioration with time suggesting the onset of cell death cascades.

Normal fibroblasts treated with 0, 25, 100, and 200 μg/mL of Ag-np recovered completely from the proliferation arrest (Figure 2A) whereas cells treated with 400 μg/mL of Ag-np recovered by the end of one month recovery period. The cell cycle analysis performed using the recovered cells showed no evidence of cell cycle arrest (Figure 2B). Untreated cells (Figure 2C) and cells treated with 25 μg/mL of Ag-np formed colonies (Figure 2D) while none of the cells treated with higher concentration of Ag-np formed colonies till the end of incubation period (Figure 2E). An increase in floating cells with time was observed at higher concentrations. The morphology of untreated cells appeared normal (Figure 2F) whereas those treated with Ag-np showed incomplete filopodia (Figure 2G). The morphology deteriorated more with time (2 weeks) giving rise to cells with no protoplasmic extensions, suggesting onset of cell death (Figure 2H). However, the nuclear morphology was well preserved suggesting no nuclear fragmentation (apoptosis).

### Up regulation of stress response genes

**Figure 3:**
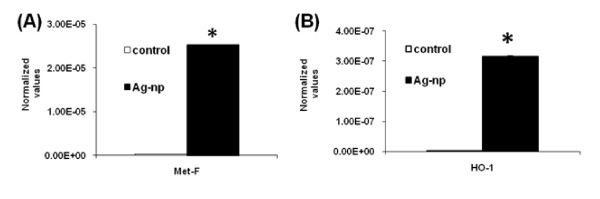
**The RT-PCR profile**. Expression profile of MT-1F (**A**) and HO-1 (**B**) in IMR-90 cells following Ag-np treatment. * p < 0.05 (n = 3). The values were normalised against the house keeping gene (18S RNA).

In a recent study, we have observed a dose and time dependent ROS production in IMR-90 and U251 cells following exposure to Ag-np [17]. Additionally, Kim *et al*. [18], using antioxidant enzymes in their study, reported that Ag-np cytotoxicity is primarily the result of oxidative stress and is independent of the toxicity of Ag+ ions. Therefore, stress response genes were selected in our study for further analysis. Real time RT-PCR confirmed an up regulation of metallothionein (MT) (Figure 3A) and hemeoxygenase-1 (HO-1) expression in Ag-np treated samples (Figure 3B).

### Intracellular distribution of Ag-np

**Figure 4:**
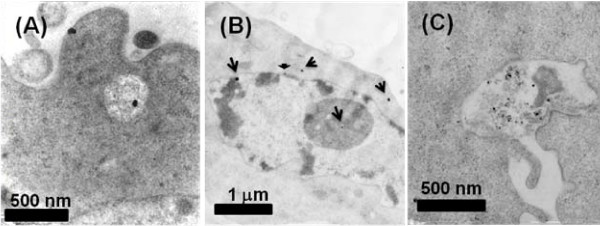
**Electron micrographs of fibroblasts treated with Ag-np**. TEM of Ag-np treated IMR-90 cells: Image shows presence of nanoparticles inside the phagosomes, near the cell membrane (**A**). Cell shows nanoparticles in the cytosol, nucleus and nucleoli. Arrow points to nanoparticles deposited. (**B**). Exocytic vesicles were observed at the cell periphery containing nanoparticles and cellular debris (**C**).

Electron micrographs revealed that Ag-np has a uniform intracellular distribution. Endosomes containing Ag-np were seen near the cell membrane (Figure 4A), suggesting nanoparticles enter the cells via endocytosis. The cytoplasm of the treated cells showed numerous endosomes with engulfed nanoparticles, autophagic vacuoles filled with structures resembling mitochondria and electron dense contents of unknown origin. Nanoparticles were observed in the nucleus of the treated cells (Figure 4B). Numerous exocytic vesicles were also observed inside the cells (Figure 4C).

### Chromosomal aberrations in Ag-np treated cells

**Figure 5:**
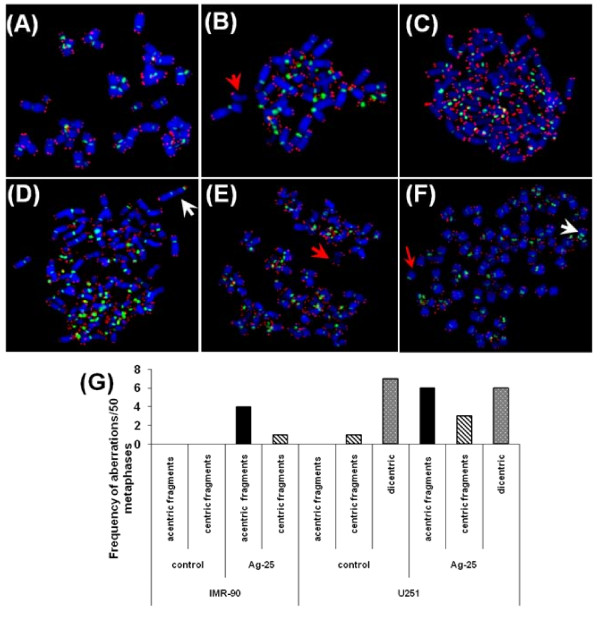
**The chromosomal aberrations in IMR-90 and U251 cells**. Metaphase spreads from the untreated cells show no apparent damage in the chromosomes (**A**), Ag-np treated IMR-90 cells show acentric and centric fragments (**B**). Arrow indicates acentric fragments. (**C**) Untreated cancer cells with no aberrations, metaphases show dicentric chromosomes in untreated cells (**D**) and treated cells. White arrow points to a dicentric chromosome. Cancer cells treated with Ag-np also show acentric fragments (**E**) and centric fragments (**F**). Red arrow points to a chromosome fragment. (**G**) Summary of the frequency of aberrations observed in Ag-np treated cells. A minimum of 50 metaphases per sample was scored for the chromosome analysis.

Ag-np treated cells were thoroughly analysed for chromosome aberrations. No significant abnormalities were observed in chromosomes of untreated fibroblasts (Figure 5A). Cells treated with high concentration of nanoparticles (50 and 100 μg/mL) did not yield enough metaphases which suggested that only a few cells were entering in to mitosis. However, fibroblasts treated with the lower dose (25 μg/mL) showed an increased percentage (10%) of aberrations characterised by the presence of acentric and centric fragments (Figure 5B). Similarly, cancer cells treated with same concentration of Ag-np showed increased chromosomal aberrations compared to control. Untreated cancer cells (Figure 5C) showed aberrations in the form of centric fragments (2%) and dicentric chromosomes (16%, Figure 5D), whereas Ag-np treated cancer cells showed many acentric (12%, Figure 5E) and centric fragments (6%, Figure 5F) and dicentric chromosomes (14%). The percentage of aberrant cells was 10% and 20% for treated normal and cancer cells respectively (Table 1). Untreated normal cells showed no aberrant cells whereas untreated cancer cells showed 16% aberrant cells. The aberrations observed in both the cell lines are represented in 5G. The number of dicentric chromosomes were comparable in control and treated groups.

**Table 1:** Summary of chromosomal aberrations observed in cancer cells and normal cells with or without Ag-np treatment

**IMR-90**									
**Ag-NP** **μg/mL**	**Metaphases analysed**	**chromosome number**	**Fragments**		**breaks**	**Fusions**		**Total aberrations** **per cell**	**Aberrant cells (%)**
								
		**(Mean ± s.e.)**	**acentric**	**centric**				**(Mean ± s.e.)**	
								
						**ring**	**dicentric**		

0	50	46 ± 0	0	0	0	0	0	0	0

25	50	46 ± 0	4	1	0	0	0	0.094 ± 0.042	10%

**U251**									

**Ag-NP** **μg/mL**	**Metaphases analysed**	**chromosome number**	**fragments**		**breaks**	**fusions**		**Total aberrations** **per cell**	**Aberrant cells (%)**
								
		**(Mean ± s.e.)**	**acentric**	**centric**				**(Mean ± s.e.)**	
								
						**ring**	**dicentric**		

0	50	93 ± 2.25	0	1	0	0	8	0.18 ± 0.062	16%

25	50	96 ± 2.46	6	3	0	0	7	0.32 ± 0.097	20%

Fifty metaphases were analysed per concentration. Higher concentrations of Ag-np did not yield sufficient number of metaphases for the analysis. Percentage of aberrant cells was expressed as number of cells which showed aberrations/number of metaphases analysed × 100.

### Calcium fluctuations in Ag-np treatment

**Figure 6:**
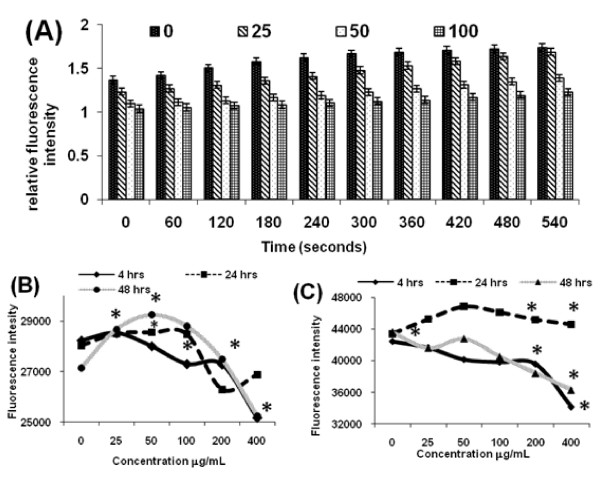
**Calcium measurements**. Calcium transients in Ag-np treated U251 and IMR-90 cells: Cells were stained with calcium binding fluorophore and subsequently activated with Ag-np (**A**). Data obtained from experiments where the U251 cells were treated with Ag-np for 4 hours and then stained with the dye (**B**). Graph denotes the calcium concentrations in Ag-np treated IMR-90 cells at 4, 24 and 48 hours. (**C**) U251 cells treated with Ag-np. * p < 0.05 (n = 4).

A typical bell shaped curve was obtained with normal cells treated with Ag-np for 48 hours. On the other hand, the kinetic study showed a drop in Ca^2+ ^concentration upon addition of Ag-np (Figure 6A). However, the fluorescence in wells treated with 25 μg/mL of Ag-np increased gradually with time. Data obtained from normal cells, which were treated with Ag-np for 4 hours showed a drop in Ca^2+ ^concentration. A gradual increase in Ca^2+ ^concentration occurred after 24 hours for Ag-np concentrations up to 100 μg/mL, above which the values fell below the control sample. However, in normal cells the Ca^2+ ^concentration stabilized by 48 hours giving a bell shaped curve (Figure 6B). The Ca^2+ ^concentration at 400 μg/mL of Ag-np was comparable to the controls in fibroblasts. In contrast, cancer cells showed a sharp drop in intracellular Ca^2+ ^ion concentration except at 24 hours (Figure 6C). Cancer cells at 24 hours showed elevated levels of Ca^2+ ^in all concentrations employed. Calcium levels were fluctuated further by 48 hours.

### Morphological changes in cells

**Figure 7:**
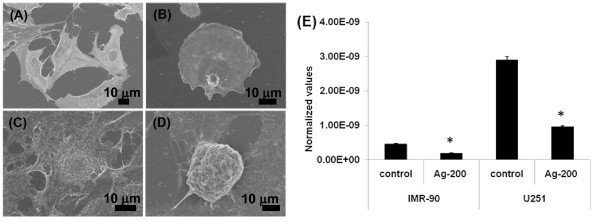
**SEM images of fibroblasts and cancer cells**: Untreated IMR-90 cells preserved their normal morphology (**A**). The Ag-np treated fibroblasts appeared more spherical and small with less cellular extensions (**B**). Untreated cancer cells showed normal morphology (**C**) whereas treated cells showed spherical cells with minimal cellular extensions and low spreading (**D**). (**E**) Filamin downregulation in normal and cancer cells as detected by RT-PCR.

Scanning electron microscopy analysis (SEM) of untreated fibroblasts revealed their normal morphology and spreading patterns (Figure 7A). Adjacent cells were connected to each other through short and slender extensions of the parent cells. No blebbing was observed in control cells. On the other hand, the Ag-np treated fibroblasts appeared more spherical, with minimum filopodia (Figure 7B). Similar pattern was observed for cancer cells. Untreated cancer cells appeared normal (Figure 7C). The spreading patterns of the Ag-np treated cancer cells were affected (Figure 7D). A few cells showed cell membrane blebbing and decreased ruffle formation. Real time RT-PCR analysis of filamin mRNA showed down regulation of filamin (Figure 7E). Cancer cells showed much lower levels of filamin compared to the normal cells.

## Discussion

Mechanism of uptake of Ag-nps is an unidentified area, which could shed light to the mechanisms of toxicity as well as potential therapeutic application of nanoparticles. In the present study, we have observed that Ag-np uptake occurs mainly through endocytosis where clathrin mediated process and macropinocytosis were involved. This observation corroborates with the electron micrographs which showed uncoated endosomes of size ~ 150 nm. The survival of cells from nanoparticle mediated damage depends on their ability to expel the nanoparticles. Previous reports have emphasised the role of exocytosis process in expelling gold nanoparticles from the cells to avoid critical loss of function [19]. Hence, exocytosis rates of Ag-nps were monitored for a better understanding of the cellular retention and expulsion of nanoparticles. Such approaches might have implications on long term deposition of nanoparticles in cells and chronic toxicity. Exclusion of Ag-np followed a slow and time dependant pattern. Though the cells were able to remove nanoparticles efficiently, the nanoparticle concentrations in cells were well within detectable limits of Ag concentration even after 48 hours of recovery, which suggests active nanoparticle retention and chances of a continuous and prolonged Ag-np mediated stress.

Metallothioneins are considered as essential biomarkers in metal-induced toxicity [20] facilitating metal detoxification and protection from free radicals [21]. Recent reports on heavy metal toxicity in Javanese medaka had shown that MT upregulation occurs in silver mediated toxicity [22]. HO-1 is an ROS sensor and a cryoprotective agent possessing antioxidant and anti-inflammatory properties. HO-1 break down heme to antioxidant biliverdin, carbon monoxide and iron under stress conditions [23,24]. Rahman *et al*. studied upregulation of oxidative stress response genes (superoxide dismutase 2, glutathione reductase 1 etc) in mouse brain following Ag-np exposure [25]. Our results substantiate existing animal studies data where upregulation of HO-1 mRNA signifies oxidative stress. Due to the antioxidant and metal detoxifying properties of HO-1 and MT, upregulation of the genes might defend the cells against the stress induced by Ag-np.

Electron micrographs have indicated endocytosis of Ag-np in to the cells and its presence in the nucleus. The exact outcome due to the nuclear deposition of Ag-np is unknown; however, it is expected to have lethal effects in DNA synthesis, DNA damage, chromosomal morphology and segregation. The deposition of metal particles inside the nucleus could affect the DNA and cell division. Genotoxic studies of titanium dioxide (TiO_2_) nanoparticles revealed dose dependant DNA damage, chromosomal aberrations and errors in chromosome segregation [26] and formation of sister chromatic exchanges [27]. Treatment with Ag-np induced the production of micronuclei (MN) [17], a marker of chromosome damage. Mroz *et al*. [28] speculated that nanoparticles and reactive oxidative species induce DNA damage, activating p53 and proteins related to DNA repair, mimicking irradiation related carcinogenesis. The observed genotoxic response, in our study, could be a consequence of oxidative damage to DNA [29].

Our experiments on Ag-np treated cells demonstrate the occurrence of calcium transients. There are substantial evidences linking oxidative stress to Ca^2+ ^transients [30] which can increase Ca^2+ ^by permeability changes of mitochondria. Ca^2+ ^ions are indispensable for cellular functions viz. cellular transport through actin dynamics and mitosis. Although Ca^2+ ^signalling occurs rapidly, a time dependant study was adopted based on the fact that nanoparticle diffusion and subsequent binding to specific receptors on cellular organelles could result in direct Ca^2+ ^transients or activation through other pathways, which will take time to initialize. Even though the binding of Ag-np to plasma membrane receptors is rapid, the rates of diffusion or endocytosis and subsequent binding with intracellular targets are relatively slow. The possibilities of continuous diffusion of nanoparticles and sustained activation of Ca^2+ ^channels are high throughout the incubation period. Moutin *et al *[31] provided evidence that Ag+ ions act on the same site as Ca^2+ ^ions, regulating the release of Ca^2+ ^from sarcoplasmic reticulum. Also, higher concentration of Ag^+ ^ion inhibited Ca^2+ ^release from the intracellular stores in a similar way as higher concentrations of Ca^2+ ^ions, giving a bell shaped curve [31]. Ag-np could release Ag+ ions through surface oxidation [17], which could trigger Ca^2+ ^fluctuations in a similar way.

Disruption of calcium homeostasis plays a major role in pathological and toxicological conditions and is an early sign of cell injury. Calcium ions have the potential to activate catabolic enzymes like phospholipase, proteases and endonuclease that further augment the toxicity [32]. Repeated calcium influx and efflux in mitochondria could result in mitochondrial membrane damage, resulting in ROS production and inhibition of ATP synthesis [32]. This report links the oxidative stress, Ca^2+ ^and ATP depletion occurred in Ag-np treated cells. Moreover, Ca^2+ ^overload in mitochondria could release apoptogenic factors such as cytochrome C, endonuclease G and other apoptosis inducing factors to the cytosol to initiate apoptosis [33]. It was observed that the rise in concentration of Ca^2+ ^ions occurred within 48 hours. Hence, it is possible that the mitochondria mediated Ca^2+ ^ions homeostasis occurred during the later stages of incubation period resulting in a delayed induction of apoptosis. It is known that an early sign of Ca^2+ ^homeostasis disruption is indicated by blebbing of plasma membrane which is a consequence of cytoskeletal injury [34]. SEM investigations to study the cytoskeletal injury revealed altered spreading patterns which may be linked to Ca^2+ ^fluctuations. Yet, the difference in response between cancer cells and normal cells towards Ca^2+ ^response and cell recovery adds on to the complexity of the mechanism.

**Figure 8:**
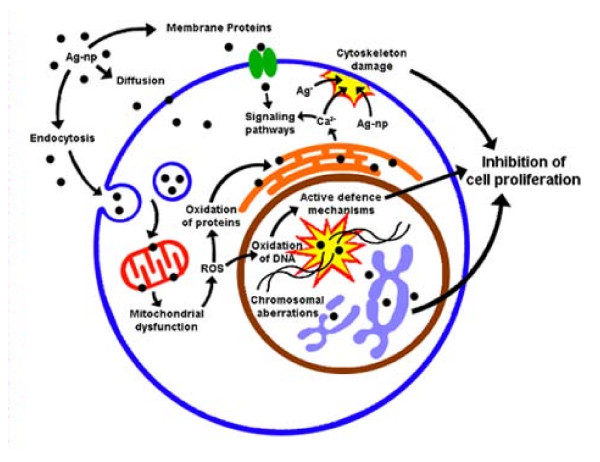
**The proposed mechanism of Ag-np toxicity based on the experimental data obtained in the present study**.

Cytoskeleton injury in most instances blocks chromosome segregation and cytokinesis. Similar patterns of cytoskeletal injury were reported in melanoma cells lacking filamin, a dimeric actin cross-linking protein [35]. The absence of filamin in cells produces unstable psuedopods (filapodia) around the cells thereby inhibiting their spreading [35]. Morphological deteriorations in cells exposed to Ag-np are possibly due to interference with structure and functions of actin cytoskeleton, which might be one of the reasons for inhibition of cell division. The cytoskeleton damage could result from calcium fluctuations and gene dysregulation. A possible mechanism of action of Ag-np which links the discussed parameters is illustrated in figure 8. The Ag-np and Ag^+ ^ions which gets released from the nanoparticles are believed to be involved in cell signalling cascades with the activation of Ca^2+ ^release that further activates catabolic enzymes and damage mitochondrial membranes. The outcome of the signalling cascades inhibits cell division.

## Conclusion

This work concludes that Ag-nps have multiple cellular targets that vary among cell types. We have identified that the major route of nanoparticle uptake is through clathrin dependent endocytosis and macropinocytosis. Also, exocytosis occurs in a time dependant manner in cancer cells. Exposure of Ag-nps resulted in chromosomal abnormalities, inhibition of proliferation, observed as failure to form colonies, and absence of recovery selectively in cancer cells, which add new hopes for preventing cancer cell metastasis. A uniform intracellular distribution of Ag-np was observed in the treated cells. Moreover, the upregulation of cryoprotective genes like HO-1 and MT-1F showed that the cellular defense mechanisms are still active in normal cells.

## Methods

### Synthesis of silver nanoparticles

Silver nanoparticles were synthesized and characterized as described in our previous publication [17]. Briefly, 1 mM silver nitrate was reduced by 0.03 gm of sodium borohydride, followed by stabilisation with soluble potato starch (0.28 gm) at 70°C. Electron micrographs showed the nanoparticle size (6-20 nm).

### Cell culture

Normal human lung fibroblasts (IMR-90) (Coriell Cell Repositories, USA) were cultured in Dulbecco's modified Eagles medium (DMEM, Sigma-Aldrich, USA) supplemented with 15% foetal bovine serum (Hyclone, USA), 2% essential amino acids and 1% each of non-essential amino acid, vitamins and penicillin-streptomycin (GIBCO, Invitrogen, USA). The cells (passage 18 ± 2) were maintained at 37°C in presence of 5% CO_2 _at log phase. Human glioblastoma cells (U251 cells) (from Dr. Masao Suzuki, National Institute of Radiological Sciences, Chiba, Japan) were maintained in DMEM supplemented with 10% foetal bovine serum (FBS, GIBCO) and 1% penicillin-streptomycin. IMR-90 cells served as a representative for normal primary cells as well as a cell type of lung origin, which is a common route of nanoparticles exposure. Ag-nps are known to localise in brain [11], thus glioblastoma cells provide a suitable model for studying interactions of nanoparticles in brain cells. Also, use of a cancer cell and normal cell allows comparison of responses between normal and cancer cells.

### Quantitation of cellular uptake of Ag-np

The nanoparticle uptake by the cells was quantitated using inductively coupled plasma-optical emission spectroscopy (ICP-OES, Perkin Elmer Optima 5300 DV). Cells were seeded at a density of 1.5 × 10^5 ^cells in T-75 flask and treated with Ag-np to achieve a final concentration of 100, 200 and 400 μg/mL of Ag-np. Higher concentrations were employed to show the stability of the nanoparticles in cell culture medium. To study the uptake mechanism of Ag-np, U251 cells were incubated with 100, 200 and 400 μg/mL of Ag-np under three different conditions. The initial step was to calculate the rate of uptake of nanoparticles when the cells were incubated for 2 hours at normal culture conditions (37°C). Endocytosis was considered as a possible mechanism of uptake based on our electron micrographs which showed endosomes with nanoparticles. Hence cells were treated with 0, 100, 200 and 400 μg/mL of Ag-np at 4°C for 2 hours to inhibit endocytosis. Low temperature treatments inhibit endocytosis in cells [19]. The transmission electron micrographs (TEM) did not show the presence of coated endosomes. This raised a suspicion that nanoparticles could be taken up by a method apart from clathrin coated pits. Hence, cells were pretreated with K^+ ^depleted medium for 30 minutes prior to nanoparticle treatment for 2 hours. This treatment blocked formation of clathrin but facilitated formation of uncoated pits (caveoli) [19]. Involvement of other uptake pathways such as macropinocytosis and caveoli dependant endocytosis were investigated by selective inhibition of the pathways in presence of specific inhibitors wortmannin [36] and nystatin [37], respectively. Analyses for macropinocytosis and caveoli mediated processes were carried out as per previous reports [36,37]. Following incubation period, medium was removed and flasks were washed 5 times with 1× phosphate buffered saline (PBS, 1^st ^Base, Singapore). Cells were harvested using trypsin - EDTA (ethylene diamine tetra acetic acid, GIBCO, Invitrogen, Grand Island, NY, USA), washed 3-4 times in PBS and resuspended in 10 mL PBS. The cell number in all tubes was adjusted to 2 million and one millilitre of the lysate was analysed after homogenization. Concentration of silver estimated from cells treated under normal conditions for 2 hours were compared with results from low temperature incubation (endocytosis blocking), K+ depleted treatment (clathrin inhibition), wortmannin (macropinocytosis inhibition) and Nystatin (caveoli inhibition).

Rate of exocytosis was studied by treating U251 cells using similar concentrations of Ag-np as described above. Following 3 hours of incubation, nanoparticles were completely washed away with buffer and further incubated for 2, 6, 24 and 48 hours in fresh medium. At the end of individual incubation period, medium and cells were collected separately, homogenised and assayed.

### Transmission electron microscopy of cells treated with Ag-np

Cells were treated with 25 μg/mL of Ag-np washed well to remove unbound Ag-np. Cells were fixed in 2.5% gluteraldehyde for 2 hours and washed in phosphate buffer. Post fixation was done in 1% osmium tetroxide for 1 hour. Cells were washed further in phosphate buffer and dehydrated in alcohol for 15 minutes each (50%, 70%, 80%, 95% and 100%). Cells were further treated with propylene oxide (30 min), propylene oxide-resin mixture (overnight) and pure resin (48 hours). Embedding was done in BEEM capsules using pure Spurr's low viscosity resin at 80°C for 48 hours. Ultrathin sections were taken using Reichert Jung Ultratome and negatively stained.

### Chromosomal Analysis by Fluorescence *in situ *hybridisation (FISH)

Cells were plated in T-75 flasks at a density of 8 × 10^5 ^and incubated with 0, 25, 50 and 100 μg/mL of Ag-np for 48 hours and metaphase spreads were prepared as explained earlier [38]. Briefly, treated cells were allowed to grow in fresh medium for 24 hours. Cells were then arrested at metaphase with 10 μL/mL karyomax colcemid solution (*Gibco*) before being subjected to hypotonic swelling in warm 0.075 M KCl and fixation with Carnoy's fixative (3:1 Methanol: Acetic Acid solution). FISH was performed on metaphase spreads using telomere and centromere specific peptide nucleic acid (PNA) probes (Applied Biosystems) labelled with Cy-3 and FITC respectively as explained earlier [38,39]. Fifty spreads were captured per sample, using Zeiss Axioplan-2 imaging (Carl Zeiss, Germany). The data were analysed using the in situ imaging software (Metasystems, Germany). Chromosomal analysis was done to detect abnormalities like chromosome breaks, fusions and abnormal segregation.

### Gene expression profile using real time-reverse transcriptase- polymerase chain reaction (RT-PCR)

Light cycler RNA amplification kit SYBR green 1 (Roche, Switzerland) was used for RT-PCR analyses as per manufacturer's instructions. Cells were treated with 200 μg/mL of Ag-nps and total RNA was isolated using RNA isolation kit (Qiagen, Germany). The concentration and integrity of RNA was measured using nanodrop spectrophotometer prior to the experiment. Primers were designed using cybrgene primer design utility. The primer (1^st ^Base, Singapore) sequence for metallothionein - 1F were 5'CCA CTG CTT CTT CGC TTC TC 3' and 5'AGG AGC AGC AGC TCT TCT TG 3' (Annealing temperature (T_A_) - 61°C) for forward and reverse primer, respectively. HO-1 gene was amplified using 5' GAG ACG GCT TCA AGC TGG TGA TGG 3' and 5' CCA CGG GGA AAG TGG TCA TGG 3' (T_A _- 61°C) as forward and reverses primers, respectively. The house keeping gene was 18s ribosomal RNA. Filamin was amplified using 5' AAGTGACCGCCAATAACGAC 3' and 5' AAGTGACCGCCAATAACGAC 3' (T_A_- 58°C) as forward and reverse primer. Amplification of the 18S rRNA was performed using 5' GTA ACC CGT TGA ACC CCA TT-3'and 5' CCA TCC AAT CGG TAG TAG CG 3' (T_A_-61°C) as forward and reverse primers, respectively.

### Intracellular calcium measurement

The Ca^2+ ^measurements were done using Fluo-2NW calcium assay kit, (Invitrogen, USA). The assay was designed to measure Ca^2+ ^transients occurring in target cells. The assay was performed as per the supplier's instructions. Ag-nps were added to the wells and incubated for different time intervals starting from 0s, 4 hours, 24 hours and 48 hours. Kinetic study was performed by prior loading of the cells with Ca^2+ ^sensors at 37°C for 30 minutes followed by incubation at room temperature for 30 minutes. Different concentrations of Ag-nps (25, 50, 100, 200 and 400 μg/mL) were added to the wells containing the dye and readings were taken immediately. End point measurements (4 hours, 24 hours and 48 hours) were taken after treatment with Ag-nps and subsequent loading with the dye. Fluorescence measurements were taken using TECAN Genios plus and BioTek Flx 800 spectrofluorometer.

### Qualitative analysis of cell morphology by SEM

Cells grown on sterile coverslips were treated with Ag-np (400 μg/mL) for 48 hours and fixed in 2.5% gluteraldehyde for 30 minutes. The coverslips were washed 5 times in Sorensen buffer and post fixed in 1% osmium tetroxide for 1 hour. Dehydration of the cells was done in ethanol series (50%, 70%, 90% and 100%). Critical point drying and platinum coatings were done as per standard SEM procedures. The slides were analysed using JEOL JSM 6701F with an accelerating voltage of 5 KV.

### Colony formation assay

This assay identifies the cell populations that are destined to die or survive following a cytotoxic drug treatment. Cells were seeded at a density of 2.5 × 10^4 ^cells in T-25 flasks and treated with 0, 25, 100, 200 and 400 μg/mL of Ag-np and incubated for 48 hours. At the end of the incubation period, medium containing Ag-np was replaced with fresh medium and formation of colony and recovery period were recorded. Two experiments were conducted for fibroblasts to ensure complete recovery from Ag-np induced stress. In the first batch of experiments fibroblasts recovered were subjected to cell cycle analysis [17]. Second set of recovered cells were assayed for the concentration of silver inside the cells.

## Authors' contributions

AR synthesised the nanoparticles, performed the experiments, analysed and wrote the manuscript. MPH and SV designed the experiments, evaluated and revised the manuscript. All authors approved the final copy of the manuscript.
